# HeAlth System StrEngThening in four sub-Saharan African countries (ASSET) to achieve high-quality, evidence-informed surgical, maternal and newborn, and primary care: protocol for pre-implementation phase studies

**DOI:** 10.1080/16549716.2021.1987044

**Published:** 2022-01-17

**Authors:** Nadine Seward, Charlotte Hanlon, Ahmed Abdella, Zulfa Abrahams, Atalay Alem, Ricardo Araya, Max Bachmann, Alemayehu Bekele, Birke Bogale, Nataliya Brima, Dixon Chibanda, Robyn Curran, Justine Davies, Andualem Beyene, Lara Fairall, Lindsay Farrant, Souci Frissa, Jennifer Gallagher, Wei Gao, Liz Gwyther, Richard Harding, Muralikrishnan R. Kartha, Andrew Leather, Crick Lund, Maggie Marx, Kennedy Nkhoma, Jamie Murdoch, Inge Petersen, Ruwayda Petrus, André van Rensburg, Jane Sandall, Nick Sevdalis, Andrew Sheenan, Amezene Tadesse, Graham Thornicroft, Ruth Verhey, Chris Willott, Martin Prince

**Affiliations:** a Centre for Implementation Science, Health Service and Population Research Department, Institute of Psychiatry, Psychology & Neuroscience, King’s College London, London, UK; b Centre for Global Mental Health, Health Service and Population Research Department, Institute of Psychiatry, Psychology & Neuroscience, King’s College London, London, UK; c Department of Psychiatry, WHO Collaborating Centre for Mental Health Research and Capacity-, Addis Ababa University, Addis Ababa, Ethiopia; d Department of Obstetrics and Gynaecology, School of Medicine, College of Health Sciences, Addis Ababa University, Addis Ababa, Ethiopia; e Alan J Flisher Centre for Public Mental Health, Department of Psychiatry and Mental Health, University of Cape Town, Cape Town, South Africa; f Department of Population Health and Primary Care, Norwich Medical School, University of East Anglia, Norwich, UK; g Faculty of Dentistry, Oral & Craniofacial Sciences, Centre for Host Microbiome Interactions, King’s College London, London, UK; h Department of Dentistry, College of Health Sciences, Addis Ababa University, Addis Ababa, Ethiopia; i King’s Centre for Global Health and Health Partnerships, School of Population Health and Environmental Sciences, King’s College London, London, UK; j University of Zimbabwe, Harare, Zimbabwe; k Psychology Department, School of Applied Human Science College of Humanities, University of KwaZulu Natal, London, UK; l Knowledge Translation Unit, University of Cape Town Lung Institute and Department of Medicine, University of Cape Town, Cape Town, South Africa; m Centre for Applied Health Research, University of Birmingham, Birmingham, UK; n Centre for Global Surgery, Department of Global Health, Stellenbosch University, Stellenbosch, South Africa; o Department of Surgery, School of Medicine, College of Health Sciences, Addis Ababa University, Addis Ababa, Ethiopia; p King’s Global Health Institute, King’s College London, London, UK; q Division of Family Medicine, School of Public Health and Family Medicine, University of Cape Town, Cape Town, South Africa; r Florence Nightingale Faculty of Nursing Midwifery and Palliative Care, Cicely Saunders Institute, King’s College London, London, UK; s King’s Health Economics, Kings College London, London, UK; t Department of Population Health Science, Kings College London, London, UK; u Centre for Rural Health, University of KwaZulu-Natal, Berea, Durban, South Africa; v Psychology Department, School of Applied Human Science College of Humanities, University of KwaZulu Natal, Berea, Durban, South Africa; w Department of Women and Children’s Health, School of Life Course Science, Faculty of Life Sciences & Medicine, King’s College London, London, UK

**Keywords:** Implementation research, implementation science, health systems strengthening, global health, Sub-Saharan Africa

## Abstract

To achieve universal health coverage, health system strengthening (HSS) is required to support the of delivery of high-quality care. The aim of the National Institute for Health Research Global Research Unit on HeAlth System StrEngThening in Sub-Saharan Africa (ASSET) is to address this need in a four-year programme, with three healthcare platforms involving eight work-packages. Key to effective health system strengthening (HSS) is the pre-implementation phase of research where efforts focus on applying participatory methods to embed the research programme within the existing health system. To conceptualise the approach, we provide an overview of the key methods applied across work-package to address this important phase of research conducted between 2017 and 2021.

Work-packages are being undertaken in publicly funded health systems in rural and urban areas in Ethiopia, Sierra Leone, South Africa, and Zimbabwe. Stakeholders including patients and their caregivers, community representatives, clinicians, managers, administrators, and policymakers are the main research participants.

In each work-package, initial activities engage stakeholders and build relationships to ensure co-production and ownership of HSSIs. A mixed-methods approach is then applied to understand and address determinants of high-quality care delivery. Methods such as situation analysis, cross-sectional surveys, interviews and focus group discussions are adopted to each work-package aim and context. At the end of the pre-implementation phase, findings are disseminated using focus group discussions and participatory Theory of Change workshops where stakeholders from each work package use findings to select HSSIs and develop a programme theory.

ASSET places a strong emphasis of the pre-implementation phase in order to provide an in-depth and systematic diagnosis of the existing heath system functioning, needs for strengthening and stakeholder engagement. This common approach will inform the design and evaluation of the HSSIs to increase effectiveness across work packages and contexts, to better understand what works, for whom, and how.

## Background

Substantial gains have been made in survival within low- and middle-income countries (LMICs), mainly through vertical programmes targeting infectious diseases including malaria, HIV and tuberculosis (TB), maternal, newborn and child conditions, and vaccine-preventable deaths [1]. However, siloed approaches to care are inefficient and undermine the aspiration of integrated people-centred care [2]. Furthermore, the epidemiological transition to greater disease burden from chronic and often multi-morbid disease, driven by increased life expectancy and globalisation of behaviours associated with unhealthy lifestyles, brings new challenges to the provision of high-quality care [1]. The coronavirus disease 2019 (COVID-19) pandemic has intensified these issues, resulting in health systems being unable to cope with the increased service use [3].

This accelerated demand and recently exposed fragility of health systems, presents challenges to the United Nations Sustainable Development Goals (SDGs). The need for Universal Health Coverage (UHC), with an implicit requirement for access to quality healthcare with financial risk protection has been amplified [4,5]. The 2017 Lancet Commission for High-Quality Health Systems, emphasises the requirement for resilient, high-quality health systems to meet the escalating demands, and to prevent against health crisis such as Ebola epidemic in west Africa [6]. The Commission describes high-quality health systems as ‘consistently delivering care that improves or maintains health, being valued and trusted by all people and responding to changing population needs’.

Achieving UHC with high-quality care is an urgent priority for health systems strengthening (HSS) in LMICs, requiring the translation of knowledge (evidence-based care) into policy and routine practice (evidence-informed care) [6,7]. HSS involves comprehensive changes to policies and regulations, organisational structures, and relationships across the health system building blocks that motivate behaviour changes among providers and patients, allowing more effective use of resources to improve healthcare across the board [8,9]. Interventions for HSS, by their very nature, improve health outcomes by providing components that influence several mechanisms, both simultaneously and also at various time points and levels of the health system levels [8].

Implementation research is a multidisciplinary approach to understand which interventions and implementation strategies work for whom, and how. This can be usefully applied to HSS by identifying and addressing barriers and opportunities to the delivery of high-quality care and testing potential solutions [10]. Of particular importance is the pre-implementation phase of research that involves a careful assessment of context to understand and address barriers to the implementation of high-quality evidence-informed health care [11]. This approach can be used to inform the development of a set of health system strengthening interventions (HSSIs) to deliver high-quality evidence-informed care, supporting the specific needs of a community and health system [9].

### The ASSET research programme

The National Health Institute of Research (NIHR) Global Research Unit on Health System Strengthening in sub-Saharan Africa (ASSET) is a four-year programme with a 10-month no-cost extension (2017–2022) that is closely aligned with the SDG goal of UHC, and the recommendations of the Lancet Commission for High-Quality Health Systems. ASSET is one of the first implementation research programmes for HSS that involves diverse care platforms, across different contexts, that also applies a common implementation science approach to the design and evaluation of HSSIs, thus allowing for comparability across settings and potential generalisability.

The aim of ASSET is to develop and evaluate effective and sustainable HSSIs, promoting consistent delivery of high-quality, people-centred care [12]. ASSET is working on three healthcare platforms in Ethiopia, Sierra Leone, South Africa, and Zimbabwe: (1) primary care for the integrated treatment of chronic conditions in adults; (2) maternal and newborn care; and (3) surgical and dental care. Eight work packages (i.e. separate research studies with work package-specific aims and objectives) use a common approach to develop context-specific HSSIs that address locally relevant and platform-specific challenges, while bringing wider system benefits. Summaries of the care platforms and associated work packages can be found in Table 1.

**Table 1.** Description of the ASSET work packages for the different healthcare platforms

Healthcare platform	Country	Specific work package (WP)
Primary health care for the integrated treatment of chronic conditions	Ethiopia	WP1. Primary care for integrated people-centred centred continuing care withchronic NCDs including diabetes and hypertension, comorbid with commonmental disorders.
	South Africa	WP4. Promoting people-centred TB care. WP5. Integrated palliative care in primary care for chronic lung disease.
	Zimbabwe	WP8. Primary care for integrated people-centred treatment with chronic NCDsincluding diabetes and hypertension, comorbid with common mental disorders.
Maternal and newborn care	Ethiopia	WP2. Integrated, people-centred maternal and newborn care across theantenatal,intrapartum, delivery and neonatal continuum. Integrated psychosocial care for perinatal women experiencingdepressionoranxiety or exposed to domestic violence is nested within this workpackage.
	South Africa	WP6. Integrated psychosocial care/support for perinatal women experiencing depression or anxiety or exposed to domestic violence.
Surgical care	Ethiopia	WP3. Increasing access to quality, equitable and affordable surgical and dental care.
	Sierra Leone	WP7. Increasing access to quality, equitable and affordable surgical care.

The ASSET programme is being conducted in two phases including the pre-implementation and piloting/rolling implementation phase. The pre-implementation phase that runs between 2017 to 2021, aims to map comprehensive care pathways of a patient’s journey through the health system including the community, different providers (e.g. private sector and non-governmental organisations), and health facilities, documenting what care is provided at what level of the health system and the associated health system bottlenecks. At the end of this phase, findings are fed back to stakeholders using focus group discussions and Participatory Theory of Change (ToC) workshops [13,14]. Stakeholders then select HSSIs and develop a programme theory. The second phase of ASSET that runs from March 2021 until January 2022 involves work packages initially piloting the set of selected HSSIs, making any necessary adaptations. This is followed by a rolling implementation phase that is an iterative process of testing, making necessary adaptations, then re-testing HSSIs. Quasi-experimental designs are used to test the effectiveness of the set of HSSIs on selected implementation and clinical outcomes and to identify factors that may influence the implementation of the proposed interventions. Integration of the interventions into routine care is being conducted in close collaboration with Ministry of Health partners in each site to support sustainability [12].

ASSET requires an extensive pre-implementation phase. A combination of different methods are used to effectively account for gaps in the provision of high-quality healthcare. The importance of taking time to engage with stakeholders who are part of the public health system cannot be underestimated, as this helps to ensure their needs and priorities are addressed and a set of HSSIs are selected to address local barriers identified for people in need of care. Another factor shaping the pre-implementation phase, is the lack/absence of routinely available data that is of sufficient quality to provide insight into key issues that need addressing (i.e. disease burden, quality of care).

**Figure 1.**
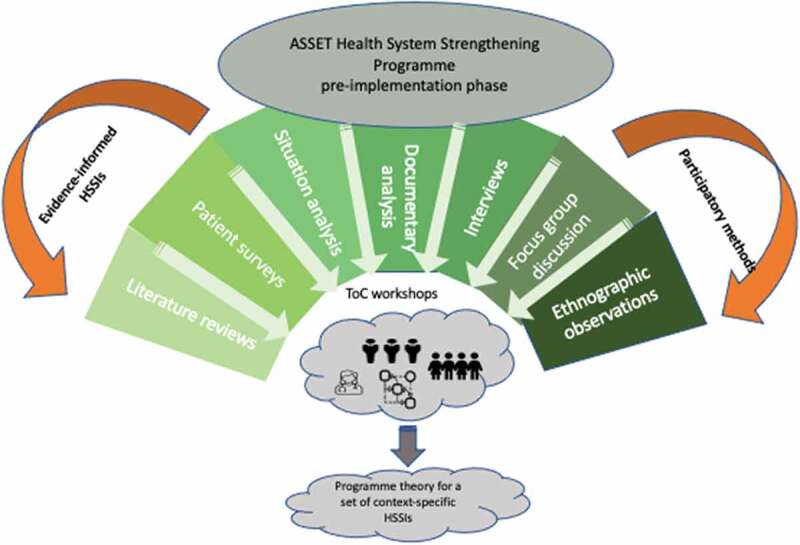
Conceptualisation of the PRE-Implementation phase of the ASSET programme

To conceptualise the pre-implementation phase of the work packages within the ASSET programme, we provide an overview of methods (Figure 1). We then review how findings are used to inform the selection and adaptation of contextually relevant HSS intervention. We also describe the synthesis of work package activities to draw conclusions on the general requirements of the health system to deliver high-quality people-centred care in LMICs [15]. To enable reproduction of individual work packages, protocols for the pre-implementation phase of ASSET are reported in Appendix 1.

The overall objectives for the pre-implementation phase (phase 1) of ASSET are:

1. To generate engagement and build relationships with stakeholders from the outset to ensure co-production and ownership of HSSIs that will survive the programme and help to generate both capacity building and sustainability.
2. To apply a mixed-methods approach (qualitative and quantitative) to evaluate the following for each of the work packages:

(I) Barriers to accessing care;
(II) Bottlenecks (critical shortage of a particular resource that results in care being blocked) in the care processes and pathways and associated outcomes;
(III) Quality of care (detection, diagnosis and treatment); and
(IV) People-centred care and its determinants.

(3) For each work package, the outputs from the different studies are used to inform the following:
  (I) ToC workshops to develop a programme theory Illustrating how and why the package of HSSIs are hypothesised to deliver valued outcomes in practice [16];
  (II) A set of HSSIs to overcome the contextual determinants of problems identified for the different care pathways at the micro, meso and macro levels; and
  (III) Selection and development of process, clinical and implementation outcome measures for the different HSSIs.

## Methods

### Study location

The eight work packages cover a range of demographic populations (i.e. with respect to age, gender, socioeconomic status, medical and social needs), located in rural, peri-urban and urban geographical settings [12]. describes the different study sites and types of publicly funded health facilities for each work package.

### Participants

The participants in the pre-implementation phase of ASSET are a combination of stakeholders, including patients and their caregivers, clinicians, managers, administrators and policy makers. ASSET also engages with important community/multi sectorial stakeholders such as non-governmental organisations and private healthcare providers.

### Study designs used within the pre-implementation phase of ASSET

To ensure the pre-implementation phase of ASSET addresses the first objective of research including engagement with stakeholders to facilitate embedding the different work packages within the existing health system, the following mixed-methods are applied across the work packages: interviews with different stakeholders, focus group discussions, and Participatory Theory of Change workshops. Other methods are also applied including literature/scoping reviews; situation analyses; cross-sectional surveys involving patients identified in healthcare facilities; cohort studies in the communities involving patients who received surgical interventions in participating healthcare facilities; documentary analyses, ethnographic observations of provision of healthcare. Table 2, Table 3 describes the objectives of for the different studies that are applied in the pre-implementation phase of ASSET.

**Table 2.** Study locations and relevant health facilities of each Work Package (WP)

Work package	Location	Public Health facilities
	**Person-centred primary healthcare for chronic conditions**	
Ethiopia (WP1)	Three districts of the Gurage Zone, Southern Nations, Nationalities and Peoples’ Region of Ethiopia.	One general hospital – staffing includes key specialists, a surgeon, an obstetrician/gynaecologist, a radiologist in addition to the staff available in primary hospitals. One Primary hospital – staffed by non-specialised doctors, integrated emergency surgical officers, health officers, midwives and nurses. 18 health centres staffed by health officers, midwives and nurses. 125 health posts staffed by community-based health extension workers.
South Africa (WP4)	Amajuba District Municipality in the province of KwaZulu-Natal. Predominately African (isiZulu) population.	Four primary healthcare facilities, staffed by nurses, and one public sector hospital, staffed by doctors and nurses. Tuberculosis treatment is limited to the public sector in South Africa and is provided free at point-of-care.
South Africa (WP5)	Cape Town Metropolitan area.	Three primary care district hospitals staffed by doctors, nurses, nursing assistants, a social worker, HIV counsellors, pharmacists and pharmacy assistants, physiotherapists, radiographers, dieticians, dentist and part-time occupational therapists. Two 24-hour community health centres with emergency units staffed by doctors, nurses, nursing assistants, a social worker, HIV counsellors, pharmacists, pharmacy assistants, a physiotherapist, part-time or full-time dietician, a part time occupational therapist, health promotion offers, dentists and dental assistants at one only. Three eight-hour community health centres with emergency rooms doctors, nurses, nursing assistants, a part-time social worker, HIV counsellors, pharmacists, pharmacy assistants, health promotors in two, part time dieticians for all, physiotherapist in one, dentist, dental assistant, and oral hygienist in one, part time rehab nurse in one and part time psychiatric nurse in one.
Zimbabwe (WP8)	The cities of Harare, Chitungwiza and Gweru.	Nine poly clinics (i.e. primary health care clinics that offered more services in terms of maternal, newborn and childcare, and HIV). The nurse in charge oversees all activities and leads the support staff consisting of community nurses, mental health nurses, midwives, HIV counselors, lay health workers, nurse aids, and pharmacy technicians. Medical doctors are not permanently present and will hold clinics on specific days in the poly clinics which also influence the composition of the clinic user population on these particular clinic days (for example HIV clinic day).
	**Maternal and newborn care**	
Ethiopia (WP2)	Three districts of the Gurage Zone, Southern Nations, Nationalities and Peoples’ Region of Ethiopia.	One general hospital – staffing includes key specialists, a surgeon, an obstetrician/gynaecologist, a radiologist in addition to the staff available in primary hospitals. One Primary hospital – staffed by non-specialised doctors, integrated emergency surgical officers, health officers, midwives and nurses. 18 health centres staffed by health officers, midwives and nurses. 125 health posts staffed by community-based health extension workers.
South Africa (WP6)	Cape Town Metropolitan area.	Four Midwife Obstetric Units (MOUs) in the Cape Town Metropolitan area staffed by antenatal care nurses, midwives, health promoters and breast-feeding counsellors.
	**Surgical care**	
Ethiopia (WP3)	Gurage and Silte Zones, Southern Nations, Nationalities, and Peoples’ Region.	The Ethiopian Health Alliance for Quality cluster (7 hospitals) co-led by a general hospital in the study site.
Sierra Leone (WP7)	Western Area of Sierra Leone including Freetown and surrounding districts.	One tertiary level government facility providing surgical care in the Western Area (Freetown and surrounding districts) that is staffed by consultant surgeons and Consultant anaesthetists and the full range of staff that you would expect at a large tertiary site. Two districts hospitals with limited surgical and anaesthetic staff – a medical officer would do the operations (general doctor with no specialist training). Six Peripheral Primary Health Units staffed by nurses only.

**Table 3.** Rational/Objectives of pre-implementation phase studies

**Method (work package(s)**	**Objectives** (not all objectives apply to each work package)
Literature review (1–8)	Collate evidence for cross-cutting issues relevant to all work packages (i.e. people-centred care, skills required for HSS, integrated primary care) to better address ASSET’s main objectives. Identify evidence-base for effective HSSIs that have potential relevance to the local context.
Situation analysis (1–8)	Appraise the national and local area level health systems context including demographic characteristics, epidemiology, policies and plans, guidelines, patient’s care pathways based on both local and national guidelines, stakeholders, and community resources. Examine routinely collected data to understand the numbers of treated patients and the outcomes of care. Use routinely available data to identify the requirements of the intervention to address the gaps in the quality of care for the different recommended care pathways. Evaluate routinely collected data including information available in Healthcare Management Information Systems (HMIS) to understand coverage, completeness, quality of process and clinical outcome indicators. This can provide insight as to the availability of data to capture process and outcome indicators to contribute to the research evaluation of the intervention. Identify requirements of local facility resources and infrastructure in meeting the current disease burden including: Healthcare workers, roles, skills and competencies; availability of equipment, medications and other treatments, and investigations; policies, guidelines and procedures, and organisation of care. Identify structural barriers to delivering evidence-based care that can occur both within a health facility (e.g. waiting times, out-of-pocket costs, availability of services) as well as externally (e.g. distance to facility, transportation, childcare).
Cross-sectional surveys of people attending at healthcare facilities (1, 2, 5, 6, 8)	Evaluate the prevalence of morbidity and comorbidity to demonstrate the added value of an integrated care approach to address exiting disease burden. Document care processes and outcomes including the proportion of cases that appropriately detected and treated to identify components of the intervention that can be used to improve quality of care. Evaluate patient knowledge and awareness of the condition/s including self-care management, help-seeking behaviour, and participation in decisions around their care to identify requirements to support patients to take a more informed and active role in their care.
Cohort study of patients identified at tertiary health care facility as having a condition requiring surgical intervention and subsequently followed up in the community (3,7)	Evaluate patient reported outcomes, satisfaction with care and associated determinants to identify requirements of the intervention to address quality of care as experienced and reported by patients (wp3, wp7). Establish prevalence of peri- and post-operative complications (wp3). Evaluate level of satisfaction with inpatient surgical care (wp3, wp7). Assess disability relating to surgical care (wp3, wp7). Identify the requirements of the intervention to address the affordability of surgical care by evaluating direct and indirect out-of-pocket costs of care for patients (wp3, wp7). Examine the burden and impact of oral/ dental conditions, and their determinants as reported by the community and oral examination (wp3). Identify the unmet dental treatment needs and investigate access to care in the community (wp3). Explore the determinants of the oral/ dental conditions and barriers to dental care attendance (wp3).
Population based community survey to identify unmet need for surgical (3, 7) and dental care (3)	Evaluate the prevalence of surgical conditions (defined as those in need of assessment and/ or care), and unmet needs for surgical intervention as reported by patients (wp3). Use verbal autopsy data to determine what proportion of deaths are potentially avertable with surgical intervention (wp3). Determine the burden of life-limiting surgical conditions in the community (wp3). Evaluate for the difference in level of impairment, pain, overall functioning and days out of work between those receiving and not receiving surgical intervention (wp3). Identify nodes for improving timely access to surgical care by describing help-seeking behaviours and pathways to and through care (wp3). Compare costs for those people with surgical conditions who did or did not receive surgical intervention. Findings can be used to gain insight into how addressing structural barriers to access might impact on residual burden related to not accessing interventions (wp3). Evaluate the willingness to pay for surgical care (wp3, wp7) to inform policy development for financing, particularly equitable co-payment structures that mitigate risk of catastrophic or impoverishing health expenditure. Examine the burden and impact of oral/ dental conditions, and their determinants as reported by the community and oral examination (wp3) and the level of unmet need for care (wp3). Explore patterns of access to dental care and barriers to care of people in the community (wp3).
Documentary analysis involving review/analysis of local guidelines, policies, Health Management Information Systems (HMIS), and clinical records and case notes (1, 2, 3, 4, 7, 8)	Compare care processes and pathways, and their variation among defined patient groups, to the ideal standards described in locally applicable guidelines/ standards or what is known based on evidence-based care. Findings can be used to identify components of the intervention to improve the quality and continuity of care by enhancing adherence to evidence-based/ guideline-based care. Identify necessary improvements to the quality and frequency of documentation for routinely available information (i.e. hard copy data, HMIS) by describing current clinical record keeping practices including quality of clinical records and compliance with current guidelines and record management. Assess any routine procedures for aggregating clinical information into HMIS data (process and outcome indicators). The above outputs can help to ensure information for relevant process and clinical and implementation outcome indicators are captured. In turn, this information will be used to evaluate the impact of the intervention (context, quality of care and its determinants).
Ethnographic observations of health care practices (1, 2, 3, 4, 6)	Document the ecology of care (i.e. the physical and social/ interpersonal environment) to identify behavioural change opportunities. Ecology is a broad construct which encompasses aspects such as respect for privacy, interactions between healthcare professionals (different cadres and hierarchies), and between staff and patients. Identify factors that influence provision of person-centred, compassionate, and respectful care. Map out care pathways to understand bottlenecks. Identify training needs and other resources required to ensure quality of care, including adherence to evidence-based guidelines and patient safety.
Semi-structured interviews and focus group discussions with patients and/or healthcare workers (1–8)	Identify barriers and enablers to correctly detect and treat relevant conditions to inform the selection of HSSIs.
	Explore the concept of person-centred care and identify barriers and enablers to providing both person-centred care and treating certain conditions associated with stigma including violence against women and TB.
	Understand perceptions of staff and patients regarding quality of care to inform the selection of HSSIs.
	Understand how care pathways that were characterised in the pre-implementation phase (i.e. review of clinical documentation) are working in practice. Identify potential issues around implementation of HSSIs including barriers associated with: acceptability and feasibility of proposed intervention; knowledge and beliefs about implementation process; implementation readiness. Investigate patient’s willingness to pay for care as well as staff’s acceptability of this measure. Explore potential health system strengthening interventions that are acceptable to all stakeholders and based on findings from other studies in the pre-implementation phase of ASSET.
Participatory Theory of Change workshop with stakeholders (1–8)	Identify components of possible HSSIs that are acceptable and feasible, necessary and sufficient to effect change towards achievement of the long-term goal. Determine appropriate sequential pathways for the above interventions to achieve valued goals, and their interactions, with a logical sequence of causality between the different components. Identify relevant process indicators and outcomes and associated contextual barriers and enablers, that in turn creates a draft framework for evaluation of the HSSIs. Enable stakeholders to come to a common understanding of what the interventions will achieve. Foster buy-in from all stakeholders with agency for implementation. Identify relevant knowledge gaps that need further attention in pre-implementation phase of ASSET.

At the end of the pre-implementation phase, data from each work package is analysed and findings from the different studies are triangulated to help substantiate and add validity to the overall findings. This process illuminates where important differences and commonalities exist between different studies such as ethnographic observations and semi-structured interviews. Findings are then used to identify a set of HSSIs to overcome the contextual barriers at the micro, meso and macro levels identified for the different care pathways.

## Details of the studies conducted within the pre-implementation phase of ASSET

### Literature review (WPs 1-8)

Individual work package teams use literature reviews to establish HSSIs that are most effective to address the relevant public health issues in the local context. As an example, work package 4 is publishing a scoping review of tuberculosis and mental health disorders and person-centred care [17]. Unpublished literature reviews are also used to inform ASSET’s cross-cutting requirements for health systems strengthening including the role of non-technical skills (e.g. clinical communication skills, leadership skills, quality improvement skills); integrated primary health care; people-centred care, and; mHealth.

### Situation analyses (WPs 1-8)

According to the World Health Organization (WHO), a situation analysis is not only a collection of facts describing the epidemiology, demography and health system status of the population, but also a comprehensive assessment of the full range of current and potential future health issues and their determinants [18]. A situation analysis therefore assesses the current disease burden and whether the health system is equipped to provide the associated high-quality care to treat the population needs.

Work packages 1–3, 6 and 7 conduct situation analyses of primary health facilities using an adapted version of the situation analysis tool developed by Programme for Improving Mental Health Care (PRIME) [19]. The PRIME situation analysis tool was originally developed to appraise district and sub-district mental health systems and services in LMICs for primary care but has broader applicability to chronic care. The ASSET programme uses the adapted PRIME tool to assess publicly available information such as existing policies and guidelines and data to determine the location and nature of the gaps between what services intend to provide compared to what is achieved in practice. The tool is also used to identify some of the critical shortages (staff, skills, knowledge) that contribute to these gaps. Of particular relevance to ASSET is using the tool to assess the availability and quality of HMIS data [19].

Work Packages 3 and 7 also use the Hospital Assessment Tool, developed by the Ethiopian Federal Ministry of Health in collaboration with the WHO and Programme in Global Surgery and Social Change, to assess secondary and tertiary care facilities for surgical care [20,21].

### Cross-sectional patient questionnaires in health facilities (WPs 1, 2, 5, 6, 8)

Patients presenting at the different health facilities are recruited consecutively when they attend for care. Following consultation, work packages teams administer validated and bespoke questionnaires to patients who present at primary and secondary health facilities. The questionnaires are used to evaluate the local health systems context including demographic characteristics, epidemiology, and patient’s care pathways based on both local and national guidelines. The clinical notes are also reviewed to identify diagnoses and management plans that are compared to the research clinical measures from the questionnaires. Patients identified as having a condition of interest, are asked additional questions including about their awareness about self-management and their involvement in decision-making and care planning. This information is critical in order to establish the extent of the care gaps and to describe parameters that can influence local HSSI development.

Although in many instances similar questionnaires are used to measure the same outcome (i.e. PHQ-9) for different work packages, in some cases work packages use questionnaires with specific relevance to their local context. The assessment tools are described in Table 4. All scales are available from the corresponding author upon request.

**Table 4.** Data collection tools and instruments used in quantitative patient surveys

Data collection tools	Platform	Work package	Country	Languages available
**Cardiovascular questionnaires**				
Respiratory symptoms: IUATLD Respiratory Questionnaire. [22]	**1**	1	Ethiopia	Amharic and English
Chronic Obstructive Pulmonary Disease – Population Screener (COPD-PS) [23].	**1**	1	Ethiopia	Amharic and English
The London Chest Activity of Daily Living scale (LCADL) [24].	**1**	5	South Africa	English
COPD Assessment Test (CAT). [25]	**1**	5	South Africa	English
African Palliative Care Associate African Palliative Outcome Scale (APOS). [26]	**1**	5	South Africa	English, Afrikaans and Xhosa, Zulu and Sotho
Memorial Symptom Assessment Scale- Short Form (MSAS-SF)	**1**	5	South Africa	English
The Australia-modified Karnofsky Performance Status Scale [27].	**1**	5	South Africa	English
**Mental illness**				
Depressive symptoms: Patient Health Questionnaire (PHQ-9). [28]	**2**	2	Ethiopia	Amharic and English
The Edinburgh Postnatal Depression Scale (EPDS) [29].	**2**	6	South Africa	English, Afrikaans and isiXhosa.
Anxiety symptoms: Generalised Anxiety Disorder-7 (GAD-7) [30].	**1,2**	1, 2	Ethiopia	Amharic and English
Shona Symptom Questionnaire of common mental disorders (SSQ-14) [31].	**1**	8	Zimbabwe	English, Shona
Alcohol and Substance use: WHO Alcohol, Smoking and Substance Involvement Screening Test (ASSIST) questionnaire [32].	**1, 2**	1, 2, 8	Ethiopia, Zimbabwe	English, Shona
World Health Organization Disability Assessment Schedule (WHODAS 2.0 12 item) [33].	**1–3**	1, 2, 3, 7, 8	Ethiopia, Zimbabwe, Sierra Leone	English, Shona
Centre for Epidemiologic Studies Depression Scale (CES-D). [34]	**1**	5	South Africa	English
The Medical Outcomes Study (MOS) Social Support Scale. [35]	**1**	5	South Africa	English
Intimate partner violence screening test [36].	**2**	2	Ethiopia	Amharic and English
Adapted Mental Health Service Satisfaction Survey (MHSSS) [37].	**2**	2	Ethiopia	Amharic and English
Trauma symptoms: Life Event Checklist (LEC) [38] and post-traumatic stress disorder checklist for DSM-V (PCL-5). [39]	**2**	2	Ethiopia	Amharic and English
A validated instrument is used to screen maternity notes for domestic violence [40].	**2**	6	South Africa	English, Afrikaans and isiXhosa.
**Surgica**l **Care**				
Patient assessment of healthcare for inpatient care (I-PAHC) questionnaire developed and validated in Ethiopia [41].	**3**	3, 7	Ethiopia, Sierra Leone	Amharic and English In Sierra Leone, translators read the questionnaire that was in English and translated to Krio for the participant.
Household economic impact of surgical care are evaluated by adapting household survey instrument used previously in the WHO SAGE study [42].	**3**	3	Ethiopia	Amharic and English
Surgeons Overseas Assessment of Surgical Need (SOSAS) questionnaire is administered using methodology adapted from a similar survey conducted in Sierra Leone. [21]	**3**	3	Ethiopia	Amharic and English
Adapted 5^th^ Edition of World Health Organisation Oral Health Survey [43].	**3**	3	Ethiopia	Amharic and English
Adapted UK (England, Wales and Northern Ireland) adult dental health survey 2009 [44].	**3**	3	Ethiopia	Amharic and English
Adapted UK Children’s dental health survey 2013 and the International Caries Detection and Assessment System – ICDAS dental caries scoring system [45].	**3**	3	Ethiopia	Amharic and English
Adapted version of the Client Service Receipt Inventory (CSRI) to examine costs associated with the surgical condition [46].	**3**	3	Ethiopia	Amharic and English
Adapted version of Willingness to Pay Survey [47].	**3**	3, 7	Ethiopia	Amharic and English
Hospital Survey on Patient Safety Culture (HSOPS) [48].	**3**	7	Sierra Leone	English In Sierra Leone, translators read the questionnaire that was in English and translated to Krio for the participant.
Household survey of economic impact of surgical care for patients discharged from main tertiary hospital. [42]	**3**	7	Sierra Leone	English In Sierra Leone, translators read the questionnaire that was in English and translated to Krio for the participant.
**Bespoke questionnaires**				
Bespoke questionnaire to collect experiences of abuse	**1**	6	South Africa	English, Afrikaans and isiXhosa.
Bespoke questionnaires to gather demographics and medical history	**1, 2**	1, 2, 5, 8	Ethiopia, Zimbabwe	English, Shona
Physical examination/ clinical assessments	**1, 2**	1, 2, 5, 8	Ethiopia, Zimbabwe, South Africa	English, Shona
Bespoke instrument to detect experiences of abuse	**2**	6	South Africa	English, Afrikaans and isiXhosa.
Bespoke questionnaires to collect information on help-seeking pathways; self-report of initial management, advice, and elicitation of patient preferences; satisfaction with care, and its outcomes; knowledge of self-care options.	**1, 2**	1, 2, 3, 8	Ethiopia, Zimbabwe	English, Shona

## Community surveys for cohort study of surgical patients recruited in health care facilities in Ethiopia and Sierra Leone

Work packages that are a part of the surgical care platform, recruit patients who are identified in participating health facilities for follow-up assessment in the community post discharge. Patients are administered questionnaires to identify peri and post-operative infection rates, unmet need for care, disability, help-seeking behaviour, satisfaction with care, household economic impact of surgery, and healthcare satisfaction. Outcomes are linked to clinical processes documented for the associated hospital admission to understand gaps in care that can be addressed with the HSSIs. Unique to WP3 in Ethiopia, is the HSS for dental care. Table 4 describes the specific surveys administered by each work package.

### Documentary analyses (WPs 1-4, 7-8)

A documentary analysis is used to identify the extent and quality of clinical documentation, through review of clinical records, guidelines, policies, and Health Management Information Systems (HMIS). Clinical records are compared to policy guidelines using methods such as process mapping, checklists, observations of patient flow, review of HMIS data, and review of clinical records using proformas. Findings are used to assess for adherence to guideline-based care or evidence-based care. Table 5 describes the relevant guidelines.

**Table 5.** Data sources and data collection instruments for documentary analysis

**Guidelines**	**Description**	**Platform** **(work package)**
Ethiopian Primary Healthcare Clinical Guideline [49].	The Ethiopian Primary Healthcare Clinical Guidelines have been contextualised from the Practical Approach to Care Kit [49]. The PHCG integrates care for all common presentations to primary health care, based on the best available evidence. PHCG promotes holistic care through integrated treatment algorithms that consider multimorbidity.	1, 2 (1, 3)
Essential drugs list and standard treatment guidelines for Zimbabwe (EDLIZ) [50].	The essential medicines list and standard treatment guidelines covers the most common health conditions in Zimbabwe and is based on the essential medicines concept. It is endorsed by the National Medicine & Therapeutics Policy Advisory Committee (NMTPAC) and was collaboratively created health care workers of all levels of the health care system. It is continuously revised and updated.	1 (8)
Ideal Clinic Policy PACK Guidelines [51]	Ideal Clinic policy promotes integrated clinical services for all patients with a view that patients receive all care by one clinician. PACK guidelines are clinical decision support tools, providing an evidence-based, comprehensive clinical approach to support the treatment of common symptoms, syndromes, diagnoses and conditions. The guides are designed for use in each consultation and starts with screening and a symptom-based approach, guides the diagnosis of common conditions, including priority chronic conditions and facilitates the routine care of the patient with one or several chronic conditions. PACK guides include PACK Child, Adolescent, Adult, Community and Home, thereby covering the life course and supporting all health workers in the primary care team [52].	1 (4)
Adult Primary Care (APC) Guidelines [53].	APC guidelines are a comprehensive clinical tool for primary care of adults 18 years or older. The guidelines were developed using approved clinical policies and guidelines issued by the National Department of Health and is intended for use by health care practitioners. APC is being implemented as part of the Integrated Clinical services Management, a key focus within the Ideal Clinic.	1 (4)
National Tuberculosis Management Guidelines [54].	The National Tuberculosis Management Guidelines provide South African department of Health’s guidance for management of TB, guidance on the management of adverse drug events and anti-retroviral initiation for patients co-infected with HIV.	1 (4)
National Infection Prevention Control Guideline for TB, MDR-TB and XDR-TB [55]	The National Infection Prevention Control guidelines for TB, MDR-TB and XDR-TB provide guidance for staff to minimise the risk of TB transmission in health settings. Infection control measures should be established to reduce risk of TB transmission to both the general population and health care personnel.	1 (4)
WHO surgical checklist [56].	The WHO Surgical Safety Checklist was developed to decrease errors and adverse events and increase teamwork and communication in surgery. The 19-item checklist has demonstrated a significant reduction in both morbidity and mortality and is now used by a majority of surgical providers around the world.	3 (3, 7)
Amajuba Mortality Report [57,58].	Summary of TB mortality trends from routine data systems in the Amajuba District Municipality.	1 (4)
Sierra Leone Early Warning Score (SLEWS)) [59]	Sierra Leone Early Warning Scoring system (SLEWS) helps to identify deteriorating patients based on a numerical scoring system given to abnormal physiological parameters.	3 (7)

### Ethnographic and structured observations of healthcare practices and context (WPs 1-4, 6)

ASSET applies both unstructured and structured ethnographic observations of clinician-patient encounters to complement quantitative methods. Unstructured observations are used extensively to understand quality of care and the broader context of patient interactions. Unstructured ethnographic observations are the best approach for exploring stigmatised conditions like TB, mental illness and domestic violence, that also captures the quality of clinician–patient interactions from a non-clinical perspective. In particular, these methods are useful for looking at issues like respect, compassion, and quality of listening. Communication of healthcare workers amongst themselves and with patients are observed to understand adherence to guideline-based care and the extent to which care is respectful and people-centred. Structured observation of clinician-patient encounters is conducted using observational checklists, including the enhancing assessment of common therapeutic factors (ENACT) rating scale for competence in elements of person-centred care [60]. Checklists are also used to help to determine the extent to which clinicians are adhering to guideline-based care.

### Semi-structured interviews and focus group discussions (WPs 1-8)

Qualitative semi-structured interviews and focus-group discussions are used by all work packages to triangulate findings with data from the quantitative surveys and observational data, and to explore perspectives of various stakeholders. Interviews are held with different groups of participants separately, allowing for frank expression of what people experience when probing around sensitive topics. Such an approach provides the human narrative component to complement the quantitative methods and understand trends in data. Table 6 describes objectives, processes and participants for the interviews and group discussions.

**Table 6.** Summary of qualitative data collection methods and samples

	Semi-structured interviews/Focus group discussions
Objectives	1. To identify health system barriers and facilitators to understand ability to: i. access treatment ii. accurately detect conditions, iii. delivery of integrated, people-centred care iv. engage patients on care pathways iv. adherence and retention in care and treatment-to-target;2. To explore the acceptability and feasibility of potential health system strengthening interventions with both patients and healthcare workers, for integrated care and how they could best be implemented to optimise care and improve outcomes.
Processes	1. Engage with clinicians to explore organisation of care, perspectives of care, pathways, components of care pathways, processes, quality, patterns of health seeking, and attitudes towards people with conditions that are known to experience stigma.2. Engage with patients on care pathways to explore experiences of living with conditions, care needs, perspectives of treatment journey, and patterns of health seeking;3. Engage with people who have not sought treatment in the formal health system to understand reasons for not doing so;4. Interviews with managers and policy makers to explore current services and interventions to support patients.5. Explore costs associated with care.
Participants	1. Primary health care workers and managers; District/zonal and regional health management; People with mental health and other NCDs diseases; Community health workers (i.e. community health workers, traditional birth attendants, religious healers, pharmacists, nurses, family physicians, NGOs); Policy makers

### Data analyses

A combination of mixed methods are used to analyse the data collected as part of the pre-implementation phase. Quantitative outcomes of interest are reported as means and proportions, accounting for clustering where appropriate. Mixed regression analysis accounting for clustering where appropriate, is used to identify determinants of quality of care (i.e. accurately detecting conditions), satisfaction with care, recovery, and risk factors for the condition in question.

Qualitative analyses use simple descriptive summaries for the outcomes of interest. Thematic framework analysis is used for in-depth interviews and focus group discussions. An inductive approach is used to analyse unstructured ethnographic observations.

### Methods to inform the piloting and rolling evaluation phases of ASSET

#### Theory of change workshops to develop a programme theory

Findings from the pre-implementation phase of ASSET are shared with the stakeholders during ToC workshops in order to elicit their ideas and priorities for quality improvement. Findings which reflect negatively on quality of care provided within a service are conveyed in such a way so that they can be shared with staff to engage in quality improvement. Confidentiality is key to this process, as is the use of patient narratives (including patient quotes). This approach helps to engage staff in constructive ways conveying how they could work differently. This shifts the quality improvement process from a culture of inspection and punishment to one of true reflection and change.

In the pre-implementation phase of ASSET, work package teams oversee between one and three ToC workshops to develop an initial programme theory. In subsequent phases of the ASSET programme, work package teams use a series of ToC workshops to adapt and refine the initial programme theory as the implementation process progresses. The result is a final programme theory that articulates pathways to change, intermediate outcomes, clinical and implementation outcomes, and underlying assumptions including contextual barriers and enablers. Each work package team invites different cadres of stakeholders to relevant meetings and workshops. Table 7 describes the ToC workshops used in each work package.

**Table 7.** ToC workshops conducted for each work package in the pre-implementation phase of ASSET

Work package	Number of workshops	Timing of workshop and relevant stakeholders involved
**Integrated primary healthcare care for chronic conditions**		
Ethiopia (WP1) Integrated care for persons with NCDs diseases, including common mental disorders	3	Workshops are held at the beginning of the pre-implementation phase that include stakeholders (Community representatives, health extension workers, primary care clinicians, secondary care clinicians, mental health professionals, and managers).One workshop is held at the end of the pre-implementation phase: national/regional level stakeholders including district, regional and national level administrators and policymakers, service user association representatives, mental health and NCDs disease clinicians and primary care clinicians.
Zimbabwe (WP8) Integrated care for persons with NCDs diseases, including common mental disorders	1	Held at the end of the diagnostic phase involving community health workers, primary health care nurses, mental health professionals, diabetes association representatives, traditional healers, patients, health service managers and policy makers.
South Africa, Cape Town (WP4) Promoting person-centred TB care	3	ToC with initial findings from the pre-implementation phase that involves the District TB Programme Coordinator, Hospital CEO, clinical and nursing management, facility managers, and Primary HealthCare (PHC) manager.ToC including reporting of additional research requested at the first workshop that included TB District Manager, Hospital Manager, Facility Managers, PHC manager, clinicians.Co-development of intervention that includes Community Health Worker Manager, Operational Managers of facilities, District Director, Nursing Managers, Ward-Based Outreach Team leaders (who supervise teams of community health workers), and PHC Supervisors.
South Africa, Cape Town (WP5) Integrated palliative care with chronic obstructive pulmonary disease	1	Held at the end of the diagnostic phase involving patients, family members, primary care physicians, palliative care physicians, respiratory physician, one representative from the department of health.
**Maternal and newborn care**		
Ethiopia (WP2) maternal and newborn care across the antenatal, intrapartum, delivery and neonatal continuum; Integration of psychosocial care for perinatal women experiencing mental health problems or exposed to domestic violence	2 3	ToC at the beginning of the pre-implementation phase that includes: community representatives, health extension workers, primary care clinicians, secondary care clinicians, and managers). Results of this workshop are shared with the surgical ToC, given the overlap in stakeholders.ToC is also held at the end of the pre-implementation phase with national/regional level stakeholders including district, regional and national level administrators and policymakers, service user association representatives, clinicians.Two ToCs are held at the beginning of pre-implementation phase: district-level participants including NGO representative and women with experience of IPV, community health extension workers and primary health care clinicians. At the first workshop there is also an expert group including mental health researchers, mental health clinicians, social workers, a psychologist with experience in adapting/delivering mental health interventions in the study site.Intervention adaptation workshop: perinatal women with experience of depression and primary healthcare workers.
South Africa, Cape Town (WP6) Integration of psychosocial care for perinatal women experiencing mental health problems or exposed to domestic violence	1	Held at the end of the pre-implementation phase involving health service managers in the Western Cape Department of Health responsible for PHC, maternal health and mental health in the City of Cape Town and its sub-districts.Key to finalising the programme theory is the continued engagement through feedback sessions at the health care facilities with stakeholders.
**Surgical care**		
Ethiopia (WP3) Surgical and dental care	3	The first ToC is held at the beginning of the pre-implementation phase includes: community representatives, health extension workers, primary care clinicians, secondary care clinicians, and managers. Results of this workshop are also shared with maternal obstetric care work package.ToC held at end of pre-implementation phase with national/regional level stakeholders including district, regional and national level administrators and policymakers, service user association representatives, clinicians.
Sierra Leone (WP7) Surgical care	2	The first ToC is held at the end of the baseline assessment of the health system, and attended by patient representatives, senior hospital managers, senior surgeons, junior doctors, anaesthetists, nurses including matrons, Primary Health Unit leads, representatives from Ministry of Health and Sanitation, local Non-Governmental Organisations. The second ToC is held a few months later and attended by senior hospital managers, senior surgeons, junior doctors, anaesthetists, nurses including matrons.

#### Implementation science methods

Implementation science theory-based determinant frameworks provide a systematic approach to the identification and description of contextual factors that are known to influence implementation outcomes, as well as key factors to consider in the design, implementation and evaluation of the HSSIs [61]. Implementation science frameworks are applied to findings from the pre-implementation phase of ASSET to help interpret the results, identify commonalities and differences across platforms and countries and ultimately help identify how context at the micro, meso and macro levels may influence the implementation of evidence-informed care.

The implementation science element of the ASSET programme is reported in a separate protocol, due to its breadth and complexity, as the ASSET diagnostic phase gets underway. Ultimately, the implementation science component of ASSET helps to determine the following: (1) whether any additional health systems strengthening interventions are required, (2) finalise process indicators and outcomes of interest in the programme theory developed in the ToC workshops, and (3) inform the design of the piloting and evaluation phases in terms of contextual factors that may influence the effectiveness of the HSSIs in delivering evidence-based and people-centred care.

#### Patient public involvement

Patients and the public were not involved in the designing/writing protocol for pre-implementation phase of ASSET. However, extensive participatory methods that involve both the patients and public are used in this phase of research to design, select and evaluate the HSSIs for ASSET.

#### Ethical considerations

All work packages have received separate ethics approval from the Research Ethics Committee at Kings College London (KCL) as well as the relevant country institutional and local government ethics review committees. See Appendix 2 for details for the different work packages.

## Discussion

HSS for universal health coverage with high-quality care requires the critical engagement with policy makers, researchers, service providers, and patients from the outset, to co-design interventions using high-quality, routinely available data that is responsive to the changing requirements of the users and health systems [62]. However, the current approach to strengthen heath systems in LMICs, is failing to meet these demands and is demonstrated by vertical programmes and academic research initiatives having little impact on broader health systems [62].

ASSET is a health system strengthening programme that involves the participatory design and evaluation of a set of contextually appropriate HSSIs across three healthcare platforms, within are eight work packages, in four countries in sub-Saharan Africa. Each work package addresses complex public health issues that are relevant to local requirements and contexts. Such a diverse programme requires a flexible approach to develop a set of HSSIs tailored to the local context.

This protocol describes how robust and extensive formative research methodologies are applied to identify limitations in the delivery of, and access to, quality care. A strong emphasis is placed on engagement of relevant stakeholders and embedding ASSET within the health systems from the onset, including people with health conditions, their carers, communities, clinicians and policy makers. It is anticipated that the use of participatory methods through group and individual consultations, and ToC workshops at various stages of the pre-implementation phase, helps to foster partnership and local ownership for the different interventions that are acceptable and feasible to implement, responding to the patient needs, that be sustained in the longer term. The COVID-19 pandemic demonstrates how ASSET has embedded itself within the health system whereby ministries have engaged with the different work packages to help manage the crisis.

A critical component of the pre-implementation work is mapping the care pathway into and through health services that allows the work we do to be people-centred, facilitating more compassionate conversations about how and why health systems fail people. We emphasise health systems as opposed to health providers because health systems are also failing people who provide the care, making it extraordinarily difficult to deliver care, let alone a respectful, people–centred approach. Mapping care pathways also helps to engage stakeholders, facilitating the co-production of HSSIs. Couching problems in systems language and using patient narratives to humanise them helps to ensure the health systems strengthening is inherently people-centred.

ASSET is also investing heavily in capacity building for HSS. Extensive training is provided in implementation science and other methodologies that invites a wide range of stakeholders both from the ASSET programme as well as the wider community. Training on the different methodologies for HSS not only ensures comparability of findings across different work packages and platforms with hopeful generalisability, but importantly increased capacity for research led HSSI within these countries.

The extensive process ASSET is undertaking in the pre-implementation phase, is in part due to the absence of high-quality data available in the HMIS that can be used to inform the requirements for HSS relevant to the local context. However, this process may have negative bearing on short-term outcomes as it puts delivery of the entire programme of work at risk where completed evaluations with follow-up of adequate duration to influence policy/ practice may not be delivered. Nevertheless, engaging in these activities is critical if HSS interventions seek to bring evidence-informed care to scale in a sustainable manner [62].

At the end of the pre-implementation phase of ASSET, it is hoped the common approach taken across different countries, care platforms and health conditions will facilitate cross platform learning and understanding of how differences in health systems and broader contextual influences shaped the development of the interventions. The overarching expectation is that by using an in-depth participatory process to engage with the stakeholders and map care pathways to and through the health system, we develop a HSS programme that can be implemented at scale that meets the needs and priorities of the local community. Ultimately it is hoped that this approach will provide people–centred high-quality care that is resilient to the changing dynamic of an aging population that can also prevent future shocks like Ebola and COVID-19.

## Supplementary Material

Supplemental Material

## Data sharing statement

No additional data available as a protocol paper

## Paper context

The need to design resilient health system strengthening interventions (HSSIs) to support high-quality care in sub-Saharan Africa is urgently required. We describe the protocol applied to the pre-implementation phase of a five-year research programme to strengthen health systems across four countries in sub-Saharan Africa. This work is providing the required evidence to develop HSSIs which are effective, sustainable and which promote consistent delivery of high-quality, people-centred care. The subsequent evaluation will involve comparison of HSSIs across diverse care platforms and contexts, facilitating concrete recommendations for wider generalisability to other LMICs on how to strengthen health systems to improve the quality of care.

## Preprint

A preprint, also known as the Author’s Original Manuscript (AOM) is available online https://www.medrxiv.org/content/10.1101/2021.01.06.20248468v1 [**doi**: 10.1101/2021.01.06.20248468].

## References

[1] Global Burden of Disease Mortality Causes of Death Collaborators. Global, regional, and national age-sex specific all-cause and cause-specific mortality for 240 causes of death, 1990-2013: a systematic analysis for the Global Burden of Disease Study 2013. Lancet. 2015;385:117–15. 25530442 10.1016/S0140-6736(14)61682-2 PMC4340604

[2] World Health Organisation. Framework on integrated, people-centred health services. 2016.

[3] Walker P, Whittaker C, Watson O, et al. The impact of COVID-19 and strategies for mitigation and suppression in low- and middle-income countries. Science. 2020;369:413–422. 32532802 10.1126/science.abc0035 PMC7292504

[4] United Nations. Transforming our world: the 2030 Agenda for Sustainable Development. 2015 [cited 2019 Oct 17]. Available from: http://www.un.org/ga/search/view_doc.asp?symbol=A/RES/70/1&Lang=E

[5] World Bank Group WHO. Tracking universal health coverage: first global monitoring report. Washington DC Geneva: The World Bank Group; World Health Organization; 2015.

[6] Kruk M, Gage A, Arsenault C, et al. High-quality health systems in the sustainable development goals era: time for a revolution. Lancet Glob Health. 2018;6:e1196–e252. 30196093 10.1016/S2214-109X(18)30386-3 PMC7734391

[7] Al-Janabi A, Al-Wahdani B, Ammar W, et al. Bellagio declaration on high-quality health systems: from a quality moment to a quality movement. Lancet. 2018. DOI:10.1016/S2214-109X(18)30372-3. 30196091

[8] Savigny Don AT. Systems thinking for Health system strengthening. Geneva: Alliance for Health Policy and Systems Research, WHO; 2009.

[9] World Health Organization. Everybody business: strengthening health systems to improve health outcomes: WHO’s framework for action. Geneva: WHO; 2007.

[10] Peters DH, Adam T, Alonge O, et al. Implementation research: what it is and how to do it. BMJ. 2013;347. DOI:10.1136/bmj.f7086 24259324

[11] Peters DTN, Adam T. Implementation research in health: a practical guide. Alliance for Health Policy and Systems Research, World Health Organization; 2013.

[12] ASSET Health System StrEngThening in Sub Saharan Africa. [cited 2020 Dec 01]. Available from: https://healthasset.org/

[13] Weiss C. Nothing as practical as good theory: exploring theory-based evaluation for comprehensive community initiatives for children and families. In: Jp C, editor. New Approaches to evaluating community initiatives: concepts, methods, and contexts. Washington DC: Aspen Institute; 1995.

[14] Vogel I. Review of the use of theory of change in international development. UK: Department for International Development; 2012.

[15] Seward N, Hanlon C, Abdulahi A, et al. HeAlth System StrEngThening in four sub_Saharan African countries (ASSET) to achieve high-quality, evidence-informed surgical, maternal and newborn, and primary care: protocol for pre-implementation phase studies. medRxiv. 2021 Jan 06;20248468. DOI:10.1101/2021.01.06.20248468. PMC8765245 35037844

[16] Andersen A. A community builder’s approach to theory of change: a practical guide to theory development. New York: The Aspen Insitute; 2004.

[17] Janse Van Rensburg A, Dube A, Curran R, et al. Comorbidities between tuberculosis and common mental disorders: a scoping review of epidemiological patterns and person-centred care interventions from low-to-middle income and BRICS countries. Infect Dis Poverty. 2020;9:4. 31941551 10.1186/s40249-019-0619-4 PMC6964032

[18] Rajan D. Situation analysis of the health sector. In: Schmets G, Rajan D, Kadandale S, editors. Strategizing national health in the 21st century: a handbook. Geneva: World Health Organization; 2016.

[19] Hanlon C, Luitel N, Kathree T, et al. Challenges and opportunities for implementing integrated mental health care: a district level situation analysis from five low- and middle-income countries. PLoS One. 2014;9:e88437. 24558389 10.1371/journal.pone.0088437 PMC3928234

[20] Iverson K, Ahearn O, Citron I, et al. Development of a surgical assessment tool for national policy monitoring & evaluation in Ethiopia: a quality improvement study. Int J Surg. 2020;80:231–240. 32198096 10.1016/j.ijsu.2020.03.025

[21] Groen R, Samai M, Petroze R, et al. Pilot testing of a population-based surgical survey tool in Sierra Leone. World J Surg. 2012;36:771–774. 22311139 10.1007/s00268-012-1448-9

[22] Burney PG, Laitinen LA, Perdrizet S, et al. Validity and repeatability of the IUATLD (1984) Bronchial Symptoms Questionnaire: an international comparison. Eur Respir J. 1989;2:940–945. 2606194

[23] Martinez F, Raczek A, Seifer F, et al. Development and initial validation of a self-scored COPD Population Screener Questionnaire (COPD-PS). Copd. 2008;5:85–95. 18415807 10.1080/15412550801940721 PMC2430173

[24] Garrod R, Bestall JC, Paul EA, et al. Development and validation of a standardized measure of activity of daily living in patients with severe COPD: the London Chest Activity of Daily Living scale (LCADL). Respir Med. 2000;94:589–596. 10921765 10.1053/rmed.2000.0786

[25] Jones P, Harding G, Berry P, et al. Development and first validation of the COPD Assessment Test. Eur Respir J. 2009;34:648–654. 19720809 10.1183/09031936.00102509

[26] Harding R, Selman L, Agupio G, et al. Validation of a core outcome measure for palliative care in Africa: the APCA African Palliative Outcome Scale. Health Qual Life Outcomes. 2010;8:10. 20100332 10.1186/1477-7525-8-10 PMC2825183

[27] Abernethy A, Shelby-James T, Fazekas B, et al. The Australia-modified Karnofsky Performance Status (AKPS) scale: a revised scale for contemporary palliative care clinical practice [ISRCTN81117481]. BMC Palliative Care. 2005;4:7. 16283937 10.1186/1472-684X-4-7 PMC1308820

[28] Kroenke K, Spitzer RL, Williams JB. The PHQ-9: validity of a brief depression severity measure. J Gen Intern Med. 2001;16:606–613. 11556941 10.1046/j.1525-1497.2001.016009606.x PMC1495268

[29] Cox J, Chapman G, Murray D, et al. Validation of the Edinburgh postnatal depression scale (EPDS) in non-postnatal women. J Affect Disord. 1996;39:185–189. 8856422 10.1016/0165-0327(96)00008-0

[30] Spitzer R, Kroenke K, Williams JB, et al. A brief measure for assessing generalized anxiety disorder: the GAD-7. Arch Internal Med. 2006;166:1092–1097. 16717171 10.1001/archinte.166.10.1092

[31] Patel V, Simunyu E, Gwanzura F, et al. The shona symptom questionnaire: the development of an indigenous measure of common mental disorders in Harare. Acta Psychiatr Scand. 1997;95:469–475. 9242841 10.1111/j.1600-0447.1997.tb10134.x

[32] WHO ASSIST Working Group. The alcohol, smoking and substance involvement screening test (ASSIST): development, reliability and feasibility. Addiction. 2002;97:1183–1194. 12199834 10.1046/j.1360-0443.2002.00185.x

[33] Ustun TB, Kostanjesek N, Chatterji S, et al. World Health Organization. Measuring health and disability: manual for WHO disability assessment schedule (‎WHODAS 2.0). 2010.

[34] Radloff L. The CES-D scale: aself-report depression scale for research in the general population. Appl Psychol Meas. 1977;1:385–401.

[35] Sherbourne CD, Stewart AL. The MOS social support survey. Soc Sci Med. 1991;32:705–714. 2035047 10.1016/0277-9536(91)90150-b

[36] Zink T, Levin L, Putnam F, et al. Accuracy of five domestic violence screening questions with nongraphic language. Clin Pediatr (Phila). 2007;46:127–134. 17325085 10.1177/0009922806290029

[37] Mayston R, Habtamu K, Medhin G, et al. Developing a measure of mental health service satisfaction for use in low income countries: a mixed methods study. BMC Health Serv Res. 2017;17:183. 28274231 10.1186/s12913-017-2126-2 PMC5343366

[38] Gray M, Litz BT, Hsu JL, et al. Psychometric properties of the life events checklist. Assessment. 2004;11:330–341. 15486169 10.1177/1073191104269954

[39] Blevins C, Weathers F, Witte T TD, et al. The posttraumatic stress disorder checklist for DSM-5 (PCL-5): development and initial psychometric evaluation. J Trauma Stress. 2015;28:489–498. 26606250 10.1002/jts.22059

[40] Abrahams Z, Schneider M, Field S, et al. Validation of a brief mental health screening tool for pregnant women in a low socio-economic setting. BMC Psychol. 2019;7:77. 31818326 10.1186/s40359-019-0355-3 PMC6902551

[41] Webster T, Mantopoulos J, Jackson E, et al. A brief questionnaire for assessing patient healthcare experiences in low-income settings. Int J Qual Health Care. 2011;23:258–268. 21531989 10.1093/intqhc/mzr019

[42] Anderson G, Ilcisin L, Kayima P, et al. Out-of-pocket payment for surgery in Uganda: the rate of impoverishing and catastrophic expenditure at a government hospital. PLoS One. 2017;12:e0187293. 29088302 10.1371/journal.pone.0187293 PMC5663485

[43] World Health Organization. Oral Health Surveys: basic Methods. 5th ed. France: World Health Organization; 2013.

[44] O’ Sullivan I, Lader D, Beavan-Seymour C, et al. Foundation Report: adult Dental Health Survey 2009 (Technical information). Report No. 2011 Mar 24.

[45] Anderson T, Thomas C, Ryan R, et al. Children’s dental health survey 2013 technical report England, Wales and Northern Ireland. Health Social Care Inf Centre hscic. 2015 Mar 19. Report No.

[46] Beecham JKM. Measuring mental health needs. In: Costing psychiatric interventions. London: Gaskell/Royal College of Psychiatrists; 1992. p. 163–183.

[47] Ochonma OG, Onwujekwe OE. Patients’ willingness to pay for the treatment of tuberculosis in Nigeria: exploring own use and altruism. Int J Equity Health. 2017;16:74. 28486981 10.1186/s12939-017-0574-2 PMC5424411

[48] Agency for Healthcare Research and Quality. Hospital survey on Patient Safety Culture. 2010. 2019. Available from: http://www.ahrq.gov/qual/patientsafetyculture/

[49] Feyissa YM, Hanlon C, Emyu S, et al. Using a mentorship model to localise the Practical Approach to Care Kit (PACK): from South Africa to Ethiopia. BMJ Glob Health. 2019;3:e001108. 10.1136/bmjgh-2018-001108 PMC6241984 30498596

[50] National Medicine Therapeutics Policy Advisory Committee. EDLIZ 2015: 7th essential medicines list and standard treatment guidelines for Zimbabwe. National Medicine and Therapeutics Policy Advisory Committee; 2015.

[51] National Department of Health. Ideal clinic South Africa. Ideal Clinic Manual Version 16. Pretoria, South Africa; 2015.

[52] Cornick R, Picken S, Wattrus C, et al. The practical approach to care kit (PACK) guide: developing a clinical decision support tool to simplify, standardise and strengthen primary healthcare delivery. BMJ Glob Health. 2018;3:e000962. 10.1136/bmjgh-2018-000962 PMC6195147 30364419

[53] Yau M, Timmerman V, Zwarenstein M, et al. e-PC101: an electronic clinical decision support tool developed in South Africa for primary care in low-income and middle-income countries. BMJ Glob Health. 2019;3:e001093–e. 10.1136/bmjgh-2018-001093 PMC6407554 30899556

[54] National Department of Health. National tuberculosis management guidelines. South Africa: Department of Health; 2014.

[55] National Department of Health. National infection prevention control guideline for TB, MDR-TB and XDR-TB. South Africa: Department of Health; 2015.

[56] Haynes A, Weiser T, Berry W, et al. A surgical safety checklist to reduce morbidity and mortality in a global population. N Engl J Med. 2009;360:491–499. 19144931 10.1056/NEJMsa0810119

[57] Hlongwa MNJ, Tshabala M. Profiling TB deaths in Amajuba District, KwaZulu-Natal province, South Africa. 2020.

[58] Hlongwa MNJ, Tshabalala. Characteristics of patients who died while on TB treatment in AMajuba District 2017-1018. Durban South Africa: Department of Health; 2020.

[59] University of Sierra Leone Teaching Hospitals Complex. Sierra Leone early warning scoring system (SLEWS). Sierra Leone: Connaught Freetown.

[60] Kohrt BA, Ramaiya MK, Rai S, et al. Development of a scoring system for non-specialist ratings of clinical competence in global mental health: a qualitative process evaluation of the Enhancing Assessment of Common Therapeutic Factors (ENACT) scale. Glob Ment Health (Camb). 2015;2. DOI:10.1017/gmh.2015.21 PMC5269630 28593049

[61] Nilsen P. Making sense of implementation theories, models and frameworks. Implement Sci. 2015;10:53. 25895742 10.1186/s13012-015-0242-0 PMC4406164

[62] English M, Irimu G, Agweyu A, et al. Building learning health systems to accelerate research and improve outcomes of clinical care in low- and middle-income countries. PLoS Med. 2016;13:e1001991. 27070913 10.1371/journal.pmed.1001991 PMC4829240

